# The pattern of amyloid accumulation in the brains of adults with Down syndrome

**DOI:** 10.1016/j.jalz.2015.07.490

**Published:** 2016-05

**Authors:** Tiina Annus, Liam R. Wilson, Young T. Hong, Julio Acosta–Cabronero, Tim D. Fryer, Arturo Cardenas–Blanco, Robert Smith, Istvan Boros, Jonathan P. Coles, Franklin I. Aigbirhio, David K. Menon, Shahid H. Zaman, Peter J. Nestor, Anthony J. Holland

**Affiliations:** a Cambridge Intellectual and Developmental Disabilities Research Group, Department of Psychiatry, University of Cambridge, Cambridge, UK; b Wolfson Brain Imaging Centre, Department of Clinical Neurosciences, University of Cambridge, Cambridge Biomedical Campus, Cambridge, UK; c German Center for Neurodegenerative Diseases (DZNE), Magdeburg, Germany; d Division of Anaesthesia, Department of Medicine, University of Cambridge, Cambridge Biomedical Campus, Cambridge, UK; e Cambridgeshire and Peterborough NHS Foundation Trust, Fulbourn Hospital, Cambridge, UK

**Keywords:** Alzheimer's disease, Down syndrome, Amyloid, PIB, PET, Dementia, Striatum, Preclinical

## Abstract

**Introduction:**

Adults with Down syndrome (DS) invariably develop Alzheimer's disease (AD) neuropathology. Understanding amyloid deposition in DS can yield crucial information about disease pathogenesis.

**Methods:**

Forty-nine adults with DS aged 25–65 underwent positron emission tomography with Pittsburgh compound–B (PIB). Regional PIB binding was assessed with respect to age, clinical, and cognitive status.

**Results:**

Abnormal PIB binding became evident from 39 years, first in striatum followed by rostral prefrontal-cingulo-parietal regions, then caudal frontal, rostral temporal, primary sensorimotor and occipital, and finally parahippocampal cortex, thalamus, and amygdala. PIB binding was related to age, diagnostic status, and cognitive function.

**Discussion:**

PIB binding in DS, first appearing in striatum, began around age 40 and was strongly associated with dementia and cognitive decline. The absence of a substantial time lag between amyloid accumulation and cognitive decline contrasts to sporadic/familial AD and suggests this population's suitability for an amyloid primary prevention trial.

## 1 Introduction

Adults with Down syndrome (DS) invariably develop senile plaques, composed of β-amyloid peptide (Aβ), indistinguishable from the histopathology of sporadic Alzheimer's disease (AD) [1], [2], and have a high risk for the development of early onset dementia [3] with estimated age-specific prevalence rates increasing from 20% to 50% in the fifties to 30% to 75% in those aged >60 years [4]. Comparable levels of amyloid can be observed in the brains of individuals with DS without dementia to those seen in typically developing individuals with AD dementia [3]. It is now believed that amyloid deposition in the typically developing population with sporadic AD can occur more than a decade before the clinical symptoms of dementia appear [5], [6].

Similar to autosomal-dominant AD, people with DS are genetically predisposed to increased amyloid accumulation; in DS, this is the result of triplication of the amyloid precursor protein gene, located on chromosome 21. DS is therefore a genetic example of Aβ overproduction, making it a highly complementary study group to the autosomally inherited forms of AD that are presently being investigated by the Dominantly Inherited Alzheimer Network (DIAN) [7] or in the large single kindred of the Alzheimer Prevention Initiative Autosomal Dominant Alzheimer's Disease study [8]. DS can yield crucial information about the generalizability of the pattern of cerebral amyloid accumulation across different genetically determined forms of AD. Furthermore, being a candidate group for amyloid primary prevention trials—particularly because DS is more prevalent than autosomally inherited AD—it is of critical importance to first understand the behavior of amyloid in the DS population so as to best optimize the timing of such studies.

Positron emission tomography (PET) imaging with ligands such as [^11^C]–Pittsburgh compound–B (PIB) enables in vivo quantification and localization of fibrillar Aβ deposits [9]. Previous amyloid PET studies have reported widespread cortical binding in people with DS after about the age of 40 years [10], [11], [12], [13], [14], [15] (Table 1). In younger individuals, imaging has generally shown an absence of amyloid binding, although some single isolated cases have been reported with focal striatal binding [12], [13], [14]. These previous studies have used large anatomic regions of interest (ROI) in relatively small numbers of participants, making a more fine-grained and systematic study of amyloid accumulation across different brain areas highly desirable. Moreover, with the exception of the pilot study [10] that was the precursor to the present work, all previous amyloid PET analyses in DS used standardized uptake value ratios of static images; the present study utilized the more robust method of calculating non–displaceable binding potential (BP_ND_) from dynamic image data. This report, therefore, characterizes in detail the evolution of PIB binding in cortical and subcortical regions of the DS brain.

## 2 Methods

### 2.1 Study design and participants

Forty-nine participants with DS aged 25–65 volunteered to take part and successfully completed the neuropsychological assessments and the imaging protocol of this study. Participants were identified via services for people with intellectual disabilities in England and Scotland, through the Down Syndrome Association or following responses to our Web site. All received an easy-to-read information pack containing a leaflet and a DVD (see: https://www.youtube.com/user/downsproject/videos). Participants were screened for any contraindications to magnetic resonance imaging (MRI)/PET scanning. All participants had previously received a clinical diagnosis of DS based on having the characteristic phenotype. In addition, 33 participants had been karyotyped as part of a pervious study, and all were confirmed to have full trisomy 21.

The study was approved by the National Research Ethics Committee of East of England and the Administration of Radioactive Substances Advisory Committee. Written consent was obtained from all adults with DS with the capacity to consent. For participants lacking the capacity to consent, the procedures set out in the England and Wales Mental Capacity Act (2005) were followed.

### 2.2 Clinical assessments

All participants were assessed for dementia using the Cambridge Examination for Mental Disorders of Older people with Down's Syndrome and Others with Intellectual Disabilities (CAMDEX-DS) informant interview, designed for diagnosing dementia in this population [16]. An experienced clinician (S.H.Z.), who was blind to the PIB status, allocated each participant into the categories of: “stable cognition,” “cognitive decline,” or “dementia.” “Dementia” was diagnosed in accordance with the International Classification of Diseases-10 (ICD-10) criteria for dementia. “Cognitive decline” diagnosis was given to participants with evidence of functional decline in one or more cognitive domains without fulfilling the full ICD-10 criteria for dementia. All participants, except three who had serve dementia and were untestable, underwent the cognitive function assessment component of the CAMDEX schedule–CAMCOG.

### 2.3 Imaging protocol

#### 2.3.1 PET using [^11^C]–PIB

[^11^C]–PIB PET data were acquired in three-dimensional (3D) mode on a GE Advance scanner (General Electric Medical Systems, Milwaukee, WI, USA). Before [^11^C]–PIB injection, a 15-minute transmission scan using rotating ^68^Ge rod sources was acquired to correct for photon attenuation. [^11^C]–PIB was produced with high radiochemical purity (>95%) and specific activity (>150 GBq/μmol). [^11^C]–PIB was injected as a bolus (median = 545 MBq, interquartile range = 465–576 MBq) through an antecubital venous catheter, and data were acquired for 90 min after injection in 58 frames (18 × 5 seconds, 6 × 15 seconds, 10 × 30 seconds, 7 × 1 minute, 4 × 2.5 minutes, and 13 × 5 minutes). For each frame, sinogram data were reconstructed using the PROMIS 3D filtered back projection algorithm [17] into a 128 × 128 × 35 image array with a voxel size of 2.34 × 2.34 × 4.25 mm^3^. Corrections were applied for random coincidences, dead time, normalization, scatter, attenuation, and sensitivity. In keeping with the pilot, feasibility work done in the planning phase of this study [18], all participants with DS tolerated the 90-minute scan without major head movement and mostly fell asleep during the acquisition. All scans were also visually inspected with none showing degradation of images due to motion. All subjects also underwent volumetric MRI scanning to facilitate ROI analysis, the details of which are included in the Supplemental Material.

### 2.4 Imaging analysis

#### 2.4.1 ROI analysis

Regional PIB analysis utilized a manually improved Brodmann atlas (based on that available in MRIcron) in Colin27 space. Several very small Brodmann areas were collapsed into larger regions to avoid ROIs of subvoxel resolution (for a list of collapsed Brodmann areas, please refer to Supplementary Table 1). The amended Brodmann atlas was standardized to the study-wise space in two steps: (1) composition of affine and nonlinear transformations, followed by (2) nearest-neighbor interpolation. Transforms were computed via deformable registration of skull-stripped study-wise and Colin27 templates using the Greedy SyN mapping approach including Gaussian regularization (sigma = 3 mm) and driven uniquely by the cross-correlation metric (window radius = 4 mm) and gradient step length of 0.15 for 100 × 100 × 80 multi-resolution iterations. Skull-stripping was performed using an iterative routine available in advanced normalization tools (ANTs).

Deep brain ROIs were calculated from the study-specific template using a fully automated approach—FIRST, available from the FMRIB software library (http://fsl.fmrib.ox.ac.uk/fsl/fslwiki/FIRST). The following subcortical structures were included: striatum (caudate nucleus and putamen), amygdala, thalamus, and hippocampus. The nucleus accumbens, brain stem, and globus pallidus were excluded from the analysis due to small size, poor FIRST segmentation, and high frequency of calcified lesions, a known feature in DS [19], respectively.

#### 2.4.2 Extracting regional PIB binding data

Dynamic PET images were realigned with statistical parametric mapping (SPM) and then averaged. The resultant mean images were rigidly coregistered with ANTs to their corresponding bias-corrected magnetisation–prepared, rapid gradient–echo MRI (MPRAGE) volume. Compositions of concatenated transformations (from PET native space to study template) were calculated and applied to PET images followed by linear interpolation. The intersection of the standardized Brodmann atlas with a ≥65% gray-matter probability mask (derived from the study template SPM segmentation) was applied to spatially normalized PET images to extract time-activity curves (TAC) for each region, which were then subjected to reference tissue input kinetic modeling. The reference tissue ROI was the superior cerebellar region constrained to ≥90% gray-matter probability. To ameliorate partial volume error from cerebrospinal fluid (CSF) contamination of the ROI signal, Gaussian smoothing was applied to the CSF segment to approximate the PET spatial resolution and hence each ROI TAC (including that of the reference tissue) was divided by 1–f_CSF_, where f_CSF_ is the average CSF fraction in the ROI. For each ROI, BP_ND_ was obtained using a basis function implementation of the simplified reference tissue model (RPM) [20]. Determination of BP_ND_ with RPM used 100 basis functions with 0.04≤ *θ*_*3*_ ≤0.6 min^−1^.

### 2.5 Establishing the stages of abnormal PIB binding

For each ROI, abnormal PIB binding was assigned to a region in the PIB-positive group with a BP_ND_ exceeding two standard deviations of the same region's mean BP_ND_ in the PIB-negative group. Grouping of “positive” and “negative” was based on the striatal BP_ND_ as discussed in the following. To understand the spread of amyloid in the brain, a cumulative rank ordering of abnormal regions was created. This was done by creating a new regional stage only when participants that had abnormal BP_ND_ for that given stage had abnormal BP_ND_ for the preceding stage. The ranking table of raw data together with a detailed description of the methodology is available in Supplementary Fig. 1. In addition to the ranking of ROIs, data were also analyzed as mean cortical BP_ND_ for the entire cortex; this analysis also included 10 healthy, age-matched (mean = 36.4, range, 24–52), non–DS controls to ensure that what was deemed to be PIB-negative in the DS population would also consider negative in the general population.

### 2.6 Statistical analysis

Regional BP_ND_ data showed a non–Gaussian distribution, when analyzed for normality with the Shapiro-Wilk test (all *P* < .01). Therefore, nonparametric tests in SPSS Statistics 21.0 (IBM, Corp.) were used for all statistical analyses. Independent group and correlation analyses were conducted using Mann-Whitney U test and Kendall's tau–b test, respectively. To facilitate comparisons across studies, effect size estimate, *r*, was calculated from the statistic's *z*-score and reported for all independent group analyses. The relationship between categorical variables was compared using chi-square or Fisher exact test for comparisons with expected frequencies below five. Plots were generated in GraphPad Prism 5.04 (GraphPad Software, Inc.), and the sigmoidal curve in Fig. 2 was fitted using a four-parameter logistic model. A significance level of .05 was used for all inferences with results reported as median together with interquartile range, unless otherwise stated.

## 3 Results

### 3.1 Defining PIB-positive and PIB-negative groups

**Fig. 1:**
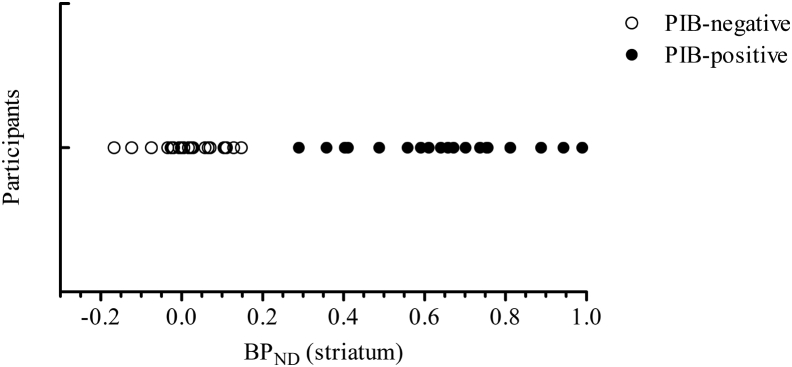
A scatter-plot representation of striatal non–displaceable binding potential for all participants, demonstrating the presence of two distinguishable populations. Classification of the two groups—PIB-positive and PIB-negative—was based on visual inspection of the data presented in this figure. Abbreviations: PIB, Pittsburgh compound–B; BP_ND_, non–displaceable binding potential.

The distribution of striatal BP_ND_ in all participants revealed a bimodal distribution (Fig. 1), a phenomenon not apparent for other regions (data not presented). Furthermore, no individual devoid of striatal binding (i.e. in the PIB-negative group) showed evidence of abnormal BP_ND_ in any other brain region (i.e. striatal binding was always present if any other brain region demonstrated binding). Therefore, subsequent analyses were conducted in two groups, PIB-negative (n = 29, open circles in Fig. 1) and PIB-positive group (n = 20, closed circles in Fig. 1), based on the bimodal distribution of striatal BP_ND_.

### 3.2 The relationship between PIB binding, age, and diagnostic status

**Fig. 2:**
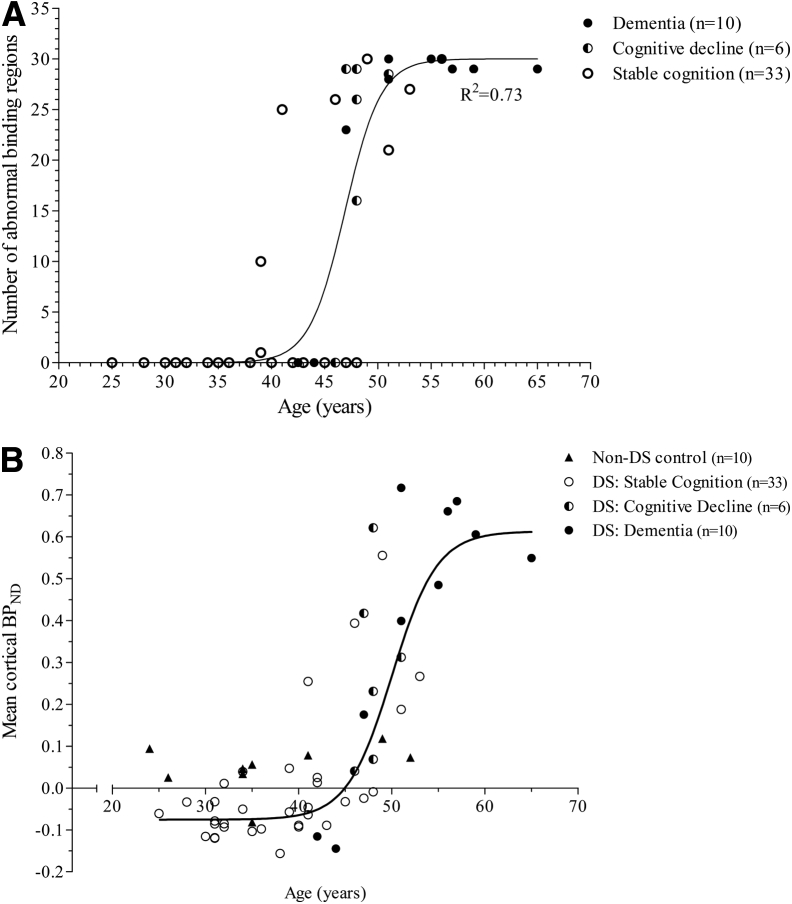
Age has a strong nonlinear relationship with (A) the number of total regions with abnormal BP_ND_ (R^2^ = 0.735) and (B) mean cortical BP_ND_ (R^2^ = 0.728) in adults with DS. The youngest participant with abnormal PIB binding was 39 years old, and all individuals in this study aged >49 years displayed both abnormal subcortical and cortical PIB binding irrespective of their dementia status. The majority in the cognitively stable group (26 of the 33) had normal PIB binding; the other seven participants showed various levels of PIB binding. The majority (five of six) in the cognitive decline group had increased binding in 16 or more regions, whereas one participant in the cognitive decline and two in the dementia group had no evidence of increased PIB binding. Regions with abnormal/increased binding are defined as those with a BP_ND_ value that exceeds two standard deviations of the mean BP_ND_ for that given region in the PIB-negative group. (B) also includes BP_ND_ data of ten age-matched (mean = 36.4; range, 24–52 years), non–DS controls that act as “true control” of negative PIB binding, demonstrating that the PIB binding observed in the PIB-negative DS group is no different to what is considered negative in the typically developing population. Please note that some data points in (A) are overlapped if two or more participants of the same age had the same number of abnormal PIB-binding regions. Abbreviations: BP_ND_, non–displaceable binding potential; DS, Down syndrome; PIB, Pittsburgh compound–B.

Demographic details of the study cohort are listed in Table 2. A strong sigmoidal relationship was apparent between age and the number of regions with abnormal binding, the latter increasing exponentially from about 40 years until plateau was reached at approximately 55 years (Fig. 2A). The same steep sigmoidal relationship was observed when mean cortical BP_ND_ was plotted as a continuous variable against age (Fig. 2B). This analysis also showed that “negative” cortical binding in the DS population was no different to what is considered negative in the typically developing, non–DS subjects.

A positive correlation with age and the number of regions demonstrating abnormal PIB binding was evident for the whole cohort (Kendall's correlation coefficient tau (49) = 0.627, *P* < .001). Diagnostic status was significantly correlated with the spatial extent of amyloid deposition (Kendall's correlation coefficient tau (49) = 0.582, *P* < .001), participants with stable cognition (median = 0 abnormal binding regions) exhibited PIB binding in significantly fewer regions in comparison with those with dementia (median = 29 abnormal binding regions, Mann-Whitney U (43) = 280.50, *P* < .001, *r* = 0.60) and those with cognitive decline (median = 27 abnormal binding regions, Mann-Whitney U (39) = 167.50, *P* < .01, *r* = 0.52). There was no significant difference in the number of abnormal binding regions between participants with cognitive decline and those with dementia (Mann-Whitney U (16) = 39.50, ns, r = 0.26). A significant negative correlation was found between CAMCOG scores and the number of abnormal binding regions in the PIB-positive group (Kendall's correlation coefficient τ (16) = −0.424, *P* < .05).

### 3.3 Stages of amyloid deposition

**Fig. 3:**
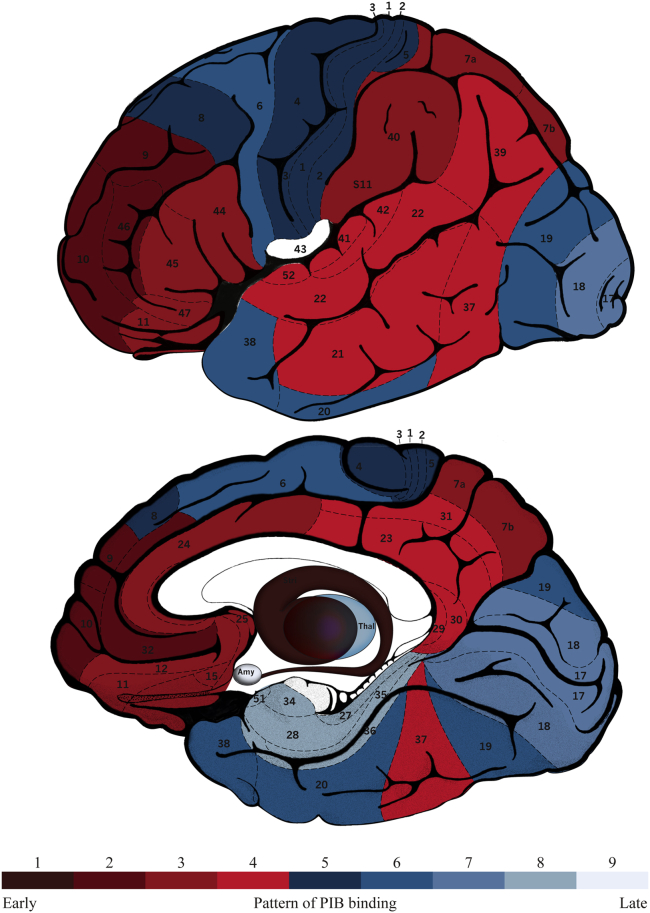
A schematic brain map of numbered Brodmann areas and subcortical regions of interest colored according to the PIB staging model, where shade 1 denotes the area affected first (i.e. the striatum) and shade 9 the area affected latest (the amygdala). Abbreviations: thal, thalamus; amy, amygdala; PIB, Pittsburgh compound–B.

The cumulative ranking of abnormal regional PIB binding is shown in Fig. 3 (for the ranking using raw BP_ND_ data, please refer to Supplementary Fig. 1). Nine stages of binding were identified. The striatum was the first region to show increased PIB binding, as 100% of individuals who were PIB-positive in one or more extrastriatal region(s) also had binding in the striatum; the second stage involved dorsal prefrontal cortex and anterior cingulate cortex; the third stage ventral prefrontal cortex and areas of the parietal lobe, including the superior parietal lobule; the fourth stage involved most of the lateral temporal cortex and the rest of the parietal lobe; the fifth primary sensory and motor areas; the sixth stage identified abnormal PIB binding in the associative visual cortex, premotor cortex, and the rest of the temporal lobe. Stage 7 was associated with amyloid in the occipital lobe. The eighth stage of the progression model involved the thalamus and parahippocampal cortex, followed by the amygdala in the final stage 9. No participants exhibited abnormal PIB binding in the hippocampus.

## 4 Discussion

This study found that PIB binding in the brains of adults with DS becomes evident from around age 40 years and increases in spatial extent in an age-dependent manner. The first brain region to become PIB-positive was the striatum, followed by PIB binding in the rostral prefrontal-cingulo-parietal regions, then caudal frontal, rostral temporal, primary sensorimotor and occipital, and finally mediotemporal regions and the rest of the basal ganglia. A critical finding of the study was that evidence of abnormal PIB binding was strongly associated with a clinical diagnosis of “dementia” or “cognitive decline” (65% of cases) without evidence of a substantial time window between the development of abnormal PIB binding and cognitive decline. This appears to represent a contrast to both sporadic AD and familial AD, where evidence suggests a latency of many years between amyloid accumulation and clinical dementia [5], [6], [21]. However, the lack of time lag between PIB positivity and cognitive decline in DS might be partially due to PIB not detecting the nonneuritic forms of amyloid plaques that are present in abundance in the DS cortex from as early as age 12 years [22]. Even so, the present study and previous investigations in sporadic and familial AD were conducted using similar methods of in vivo amyloid PET imaging, and thus have yielded findings of the estimated time course from amyloid deposition to cognitive decline that are comparable with the results of the present study.

The distribution of PIB binding in DS as shown for this study is congruent with previous findings in the DIAN cohort [21]and in sporadic AD [9]. This combined evidence suggests that amyloid accumulation, once established, is likely to be similar across different forms of AD and further supports the generalizability of the amyloid PET findings. There might, however, be a discrepancy in the temporal evolution of PIB binding between sporadic and genetic AD with respect to early striatal binding. Striatal binding was the earliest feature in this series and was universal if participants had abnormal binding in any other brain region; this replicates findings in both DS [12], [13], [14] and in familial forms of AD (amyloid precursor protein and presenilin-1 mutations carriers) [23], [24], [25], [26]. In sporadic AD, pathologic studies [27], [28] and amyloid imaging [29] have demonstrated extensive amyloid deposition in the striatum of virtually all demented AD patients. Striatal amyloid together with cortical amyloid has been also reported in cognitively normal presymptomatic individuals [29], [30]. Studies directly examining whether there may be a “striatum-only” earlier phase of PIB binding in sporadic AD are, however, lacking at present. In general, pathologic studies in sporadic disease have tended to suggest that striatal amyloid is not a very early feature [27], [28], although this region traditionally has not been intensively researched in AD. The current findings combined with those in familial AD suggest that a pathologic re-examination of this region is justified. Turning to the nature of striatal amyloid, past studies have shown that striatal plaques in sporadic AD are mainly of nonneuritic type [31], [32], although cored and neuritic plaques have also been reported [30], [33]. Post mortem studies in DS [31], [34] and in familial AD [35] have also identified mainly nonneuritic striatal amyloid with rare reports of neuritic plaques only in cases with severe cortical amyloidosis. PIB is known to bind to nonneuritic—as well as neuritic and cored—plaques [36]; therefore, the findings in this DS cohort are compatible with striatal amyloid being nonneuritic in nature. Immunohistochemistry studies have shown that striatal plaques are predominantly, or entirely, composed of Aβ1–42 and Aβ1–43 [31], [34]. Familial forms of AD and DS share overproduction of amyloid as the proposed mechanism of amyloid deposition [37], whereas decreased clearance might be more significant in sporadic AD [38]. Therefore, it might be that the cellular milieu of the striatum is particularly vulnerable to early amyloid accumulation under the conditions of overproduction.

A strength of the present study was that it showed the relative change in amyloid binding across the adult life span (from 25 to 65 years) in an ultra high-risk population for AD. As expected, higher PIB binding levels were observed in participants with higher age, lower cognitive performance on neuropsychological assessment, and in those with a diagnosis of dementia. The data showed that although comparison of the number of regions with increased PIB binding tended to separate people with dementia from those with stable cognition, it was unable to sufficiently distinguish between individuals with cognitive decline and those with dementia. This is consistent with a wealth of evidence that plaque pathology has already reached a plateau with the first signs of cognitive decline and then remains relatively stable throughout the duration of the clinical disease [6]. Interestingly, in contrast to such findings, some participants in this study had cognitive decline or dementia without having reached the plateau distribution of PIB binding (Fig. 2) possibly related to reduced cognitive reserve in the DS population. Two participants diagnosed with dementia in the cohort showed no PIB binding. Similar findings have been reported by the Alzheimer's Disease Neuroimaging Initiative PET core, showing that 10%–20% of their clinically diagnosed AD subjects are PIB-negative [39] with the suggestion that these could represent the presence of non-AD dementia [40]. Although the two apparently demented PIB-negative subjects in the present series might also represent non-AD pathology, it is probably more likely a reflection of the occasional difficulty in making an accurate early dementia diagnosis in the DS population. This is particularly exacerbated by the presence of underlying intellectual disability, frequent lack of information about individual's premorbid level of functioning, and difficulties in communication, and therefore heavy reliance on informant opinion [41].

This study has a number of limitations. A sequence of amyloid deposition in the DS population is characterized from a cross-sectional data set. Although this approach is the norm in post mortem–based pathologic staging systems, a longitudinal study in this population is essential to validate the reported findings. Furthermore, diagnosing dementia in people with intellectual disabilities (including DS) is a particular challenge with an acknowledged risk of uncertainty [41]; albeit to a lesser degree, this is also true, however, in sporadic AD. The CAMDEX–DS has been validated as a reliable tool for assessing clinical dementia in people with DS [16], which in combination with an amyloid PET scan should increase the confidence of dementia being of Alzheimer's type in DS.

Several trials to date, aimed at preventing cognitive decline in AD with anti-amyloid therapies, have been unsuccessful [42]. These studies have all targeted patients with an established amyloidosis. Primary prevention trials, in contrast, are arguably the ultimate test of anti-amyloid therapies—if such strategies were ever to work, then preventing amyloid from ever accumulating would surely be more effective than its removal. Primary prevention trials are not feasible in sporadic disease, and even in individuals with autosomal dominant mutations, the long latency between amyloid accumulation and cognitive decline would mean a primary prevention trial would have to run for many years, which would be costly and logistically difficult. The present study, however, suggests that DS is ideal for a primary prevention study for two reasons. First, it demonstrated that conversion from amyloid negative to positive occurs within a fairly narrow age window in the early forties. Second, the data (Fig. 2) indicate that there is a relatively short latency between amyloid accumulation and cognitive decline in DS—presumably related to reduced cognitive reserve as a consequence of the developmental disorder. There is also the recruitment advantage of DS being far more common than autosomal dominant forms of AD. The impact of a primary prevention trial in DS could be immense. If successful, it could eradicate dementia in DS. Moreover, the insights that such a trial could yield, whether positive or negative, would likely have a profound influence on the prioritization of anti-Alzheimer strategies more broadly.

**Research in context**

1. Systematic review: Literature review using PubMed identified six cross-sectional investigations of amyloid in people with Down syndrome (DS), reporting amyloid in frontal, parietal, and temporal lobes, cingulate cortex, striatum and thalamus. However, these reports are limited in anatomic detail and have not assessed the temporal evolution of amyloid binding in DS, thus leaving a gap in the knowledge that would be of great benefit in the development of prevention strategies for Alzheimer's disease.
2. Interpretation: Our data show that amyloid accumulation begins in the striatum in people with DS and progresses in a predictable manner. A key finding was the narrow window between amyloid accumulation and cognitive decline suggesting that a primary prevention anti-amyloid trial in DS could be feasible.
3. Future directions: Further studies are needed to validate this amyloid accumulation model in a larger data set and to establish its potential as a biomarker and tool for assessing the efficacy of amyloid-targeting agents.

## Figures and Tables

**Table 1:** Previous amyloid PET studies in individuals with DS and the main findings of amyloid binding

Study	Amyloid agent	Participants with DS	Age range (y)	Youngest amyloid positive participant	Methods	Regions with increased amyloid binding in DS
(Landt et al. 2011) [10]	^11^C–PIB	8	25–59	45	Scan 0–90 min after injection, BP_ND_ estimated with cerebellum as reference region, region-specific positive-binding thresholds	5/8: Anterior and posterior cingulate, calcarine, prefrontal, superior parietal. Hippocampus (2/8).
(Sabbagh et al. 2011) [12]	^18^F-Florbetapir	1	55	NA	Scan 50–60 min after injection, SUVR estimated using cerebellum reference region	Frontal, temporal, parietal, anterior and posterior cingulate, precuneus, striatum, thalamus
(Handen et al. 2012) [13]	^11^C–PIB	7	20–44	38	Scan 40–60 min after injection, SUVR using cerebellum reference region; region-specific positive-binding thresholds	Striatum (2/7); frontal, anterior cingulate, precuneus/posterior cingulate, lateral temporal lobe (1/7)
(Hartley et al. 2014) [14]	^11^C–PIB	63 (none with symptoms of dementia)	30–53	NA	Scan 50–70 min after injection, SUVR using subcortical white matter and cerebellum reference region, region-specific positive-binding thresholds	Striatum (21/63); anterior cingulate (13/63); precuneus, frontal and lateral temporal cortex (12/63); parietal cortex (8/63)
(Jennings et al., 2015) [15]	^18^F-Florbetaben	39	40–56	40–44	Scan 100–120 min after injection, SUVR using cerebellum reference region, composite region positive-binding threshold	Composite region (frontal, lateral temporal, anterior and posterior cingulate, parietal, occipital): 1/14 (40–44 y); 6/15 (45-49 y); 7/10 (≥50 y)

Abbreviations: PET, positron emission tomography; DS, Down syndrome; BP_ND_, non–displaceable binding potential; NA, not applicable; ^11^C–PIB, ^11^C–labeled Pittsburgh Compound–B; SUVR, standardized uptake value ratio.

NOTE. Amyloid agents refer to amyloid-specific PET radiotracers:^11^C–PIB and two^18^F–labeled amyloid radiotracers (Florbetapir and Florbetaben). A study by Nelson et al. [11] utilized a mixed amyloid/tau tracer and was thus not investigated in detail.

**Table 2:** Study demographics

Demographics	PIB-positive (n = 20)	PIB-negative (n = 29)	*P* value (effect size, *r*)
Age, y	50 (39–65)	36 (25–48)	<.0001∗ (−0.72)
Female	8 (40)	15 (52)	.419†
Dementia	8 (40)	2 (7)	<.01‡
Cognitive decline	5 (25)	1 (3)	<.05‡
CAMCOG score (max 109)	74 (17–95)	76 (38–102)	.406∗ (0.12)

Abbreviation: PIB, Pittsburgh compound–B.

NOTE. Data are shown as median (absolute range) or number (%). All participants had mild-to-moderate learning disability.

∗ Mann-Whitney U test.

† Chi-squared test.

‡ Fisher exact test.
