# Supplementary material for: The pattern of amyloid accumulation in the brains of adults with Down syndrome
Source: Alzheimers Dement. 2016 May;12(5):538–45. doi: 10.1016/j.jalz.2015.07.490 (PMC4867786; doi:10.1016/j.jalz.2015.07.490)
Supplement: Supplementary Material [file mmc1.docx]

**SUPPLEMENTARY MATERIAL**

**MRI acquisition**

To facilitate a detailed PIB ROI analysis, all participants also had an anatomical MRI scan on a Siemens Verio 3 Tesla scanner with 12 channel head coil (Siemens AG, Erlangen, Germany), using the 3D T_1_–weighted magnetisation–prepared, rapid gradient–echo (MPRAGE) pulse sequence with the following parameters: repetition time / echo time / inversion time / flip angle = 2300ms/ 2.98ms/ 900ms/ 9°, 256x240x176 matrix dimensions, and 1x1x1 mm^3^ voxel size. Receiver bandwidth and echo spacing were 240 Hz/pixel and 7.1 ms, respectively. Note that parallel acceleration was not enabled. In addition, whole–brain, T_2_–weighted, half–Fourier acquisition, single–shot turbo spin echo (HASTE) scans were acquired to assess for vascular pathology and incidental lesions. Sedation was not used during MRI or PET scanning.

**MRI preprocessing**

MPRAGE images were cropped using the ‘dcm2nii’ tool in MRIcron (available freely from http://www.mccauslandcenter.sc.edu/mricro/mricron/) and were then radio–frequency bias corrected, spatially normalised, and segmented into six tissue classes using SPM12b (available from http://www.fil.ion.ucl.ac.uk/spm; Wellcome Trust Centre for Neuroimaging, University College London) with default settings. Subsequently, a study–wise space was calculated from SPM bias–corrected T_1_–images using a parallel routine based on the diffeomorphic Greedy–SyN transformation model available as part of the Advanced Normalization Tools package (ANTs v1.9.4, available freely from http://stnava.github.io/ANTs/). The standardisation pipeline consisted of eight nonlinear warping runs – driven by fast cross–correlation minimisations – each performed over three resolution levels: 90 iterations at the coarsest level, 30 at the next coarsest, and 90 at full resolution. Template update step length was set to 0.1. Finally, warped images were linearly interpolated, scaled, and averaged to produce a study template.

**Supplementary Table 1 List of collapsed Brodmann areas with respective anatomical locations**

| **Anatomical location** | **Collapsed Brodmann areas** |
| --- | --- |
| Primary somatosensory and motor cortex | Brodmann 1, 2, 3, 4 |
| Ventro–medial prefrontal cortex | Brodmann 11, 12, 13, 14, 15, 25 |
| Superior temporal gyrus | Brodmann 22, 41, 42, 52 |
| Posterior cingulate and precuneal area | Brodmann 23, 29, 30, 31 |
| Parahippocampal cortices | Brodmann 27, 28, 34, 35, 36, 51 |

| **Region of interest** | **PIB–positive participants (n=20)** | | | | | | | | | | | | | | | | | | | | **PIB–negative participants (n=29)** | | |
| --- | --- | --- | --- | --- | --- | --- | --- | --- | --- | --- | --- | --- | --- | --- | --- | --- | --- | --- | --- | --- | --- | --- | --- |
|  | **P1** | **P2** | **P3** | **P4** | **P5** | **P6** | **P7** | **P8** | **P9** | **P10** | **P11** | **P12** | **P13** | **P14** | **P15** | **P16** | **P17** | **P18** | **P19** | **P20** | Mean | Stdev | Mean+2Stdev |
| Hippocampus | -0.10 | 0.05 | 0.12 | -0.03 | 0.09 | 0.10 | 0.06 | -0.04 | 0.09 | 0.13 | 0.00 | 0.07 | 0.09 | -0.01 | 0.05 | 0.18 | 0.14 | 0.16 | 0.12 | 0.13 | 0.01 | 0.10 | **0.20** |
| Amygdala | -0.06 | -0.04 | 0.04 | -0.02 | 0.04 | 0.03 | 0.07 | 0.03 | 0.06 | 0.09 | 0.06 | 0.15 | 0.10 | 0.07 | 0.13 | 0.06 | 0.19 | 0.11 | 0.17 | 0.09 | -0.06 | 0.06 | **0.06** |
| Parahippocampal cortices (BA 27,28,34–36,51) | -0.15 | -0.02 | 0.03 | -0.04 | -0.01 | 0.00 | 0.05 | -0.03 | 0.07 | 0.03 | 0.04 | 0.21 | 0.08 | 0.08 | 0.12 | 0.09 | 0.19 | 0.15 | 0.13 | 0.16 | -0.09 | 0.07 | **0.06** |
| Thalamus | 0.13 | 0.14 | 0.35 | 0.07 | 0.31 | 0.28 | 0.60 | 0.23 | 0.24 | 0.19 | 0.39 | 0.26 | 0.19 | 0.07 | 0.21 | 0.40 | 0.42 | 0.25 | 0.47 | 0.35 | 0.10 | 0.07 | **0.24** |
| Primary visual cortex (BA17) | -0.05 | -0.05 | -0.06 | -0.03 | 0.02 | -0.05 | -0.04 | -0.02 | 0.02 | 0.35 | 0.03 | 0.00 | 0.11 | 0.25 | 0.28 | 0.36 | 0.12 | 0.00 | 0.05 | 0.51 | -0.07 | 0.03 | **-0.01** |
| Secondary visual cortex (BA18) | -0.08 | 0.02 | -0.08 | 0.09 | 0.09 | -0.02 | -0.05 | 0.12 | -0.03 | 0.35 | 0.03 | 0.00 | 0.18 | 0.32 | 0.38 | 0.32 | 0.26 | 0.04 | 0.08 | 0.62 | -0.08 | 0.04 | **0.00** |
| Temporopolar area (BA38) | -0.17 | -0.02 | -0.06 | -0.03 | -0.09 | -0.01 | 0.04 | 0.23 | 0.20 | 0.08 | 0.10 | 0.45 | 0.29 | 0.30 | 0.17 | 0.35 | 0.35 | 0.39 | 0.16 | 0.46 | -0.11 | 0.07 | **0.02** |
| Pre-motor area (BA6) | -0.04 | 0.08 | 0.12 | 0.10 | 0.11 | 0.25 | 0.23 | 0.37 | 0.27 | 0.32 | 0.30 | 0.61 | 0.68 | 0.42 | 0.37 | 0.42 | 0.76 | 0.37 | 0.38 | 0.72 | -0.01 | 0.06 | **0.12** |
| Inferior temporal gyrus (BA20) | -0.14 | -0.03 | -0.02 | 0.01 | 0.02 | 0.07 | 0.14 | 0.30 | 0.18 | 0.18 | 0.21 | 0.57 | 0.45 | 0.32 | 0.37 | 0.49 | 0.44 | 0.51 | 0.40 | 0.56 | -0.09 | 0.07 | **0.04** |
| Associative visual cortex (BA19) | -0.06 | 0.12 | -0.04 | 0.20 | 0.16 | 0.08 | -0.09 | 0.20 | 0.06 | 0.38 | 0.12 | 0.19 | 0.36 | 0.27 | 0.50 | 0.44 | 0.62 | 0.24 | 0.35 | 0.60 | -0.07 | 0.06 | **0.05** |
| Primary somatosensory and motor cortex (BA1–4) | -0.03 | 0.04 | 0.15 | 0.12 | 0.14 | 0.26 | 0.25 | 0.27 | 0.22 | 0.47 | 0.22 | 0.50 | 0.45 | 0.39 | 0.19 | 0.36 | 0.61 | 0.46 | 0.40 | 0.40 | -0.01 | 0.07 | **0.13** |
| Somatosensory association cortex (BA5) | 0.10 | 0.10 | 0.25 | 0.29 | 0.23 | 0.66 | 0.70 | 0.50 | 0.51 | 0.58 | 0.58 | 1.13 | 0.87 | 0.67 | 0.45 | 0.91 | 1.09 | 0.82 | 0.81 | 0.77 | -0.01 | 0.13 | **0.25** |
| Dorsal frontal area (BA8) | -0.04 | 0.19 | 0.28 | 0.18 | 0.15 | 0.59 | 0.39 | 0.70 | 0.53 | 0.40 | 0.61 | 1.04 | 0.93 | 0.75 | 0.70 | 0.76 | 0.90 | 0.76 | 0.53 | 1.26 | 0.00 | 0.09 | **0.19** |
| Middle temporal gyrus (BA21) | -0.09 | 0.03 | 0.00 | 0.14 | 0.11 | 0.25 | 0.27 | 0.43 | 0.23 | 0.31 | 0.33 | 0.66 | 0.62 | 0.48 | 0.46 | 0.59 | 0.65 | 0.62 | 0.52 | 0.82 | -0.07 | 0.06 | **0.05** |
| Superior temporal gyrus (BA 22,41,42,52) | -0.02 | 0.02 | -0.03 | 0.18 | 0.19 | 0.31 | 0.38 | 0.48 | 0.21 | 0.48 | 0.28 | 0.78 | 0.63 | 0.57 | 0.50 | 0.63 | 0.74 | 0.72 | 0.58 | 0.85 | -0.06 | 0.05 | **0.04** |
| Posterior cingulate and precuneal area (BA 23,29–31) | 0.06 | 0.03 | 0.13 | 0.26 | 0.25 | 0.42 | 0.51 | 0.49 | 0.37 | 0.52 | 0.57 | 1.00 | 0.91 | 0.83 | 0.67 | 1.06 | 1.16 | 0.77 | 0.71 | 0.87 | -0.01 | 0.08 | **0.16** |
| Fusiform gyrus (BA37) | -0.08 | 0.09 | 0.00 | 0.20 | 0.10 | 0.12 | 0.09 | 0.35 | 0.16 | 0.33 | 0.18 | 0.44 | 0.47 | 0.31 | 0.44 | 0.47 | 0.63 | 0.41 | 0.44 | 0.62 | -0.05 | 0.05 | **0.06** |
| Angular gyrus (BA39) | -0.05 | 0.19 | 0.06 | 0.39 | 0.14 | 0.28 | 0.23 | 0.43 | 0.28 | 0.49 | 0.38 | 0.63 | 0.70 | 0.52 | 0.53 | 0.68 | 0.83 | 0.68 | 0.76 | 0.70 | -0.06 | 0.07 | **0.07** |
| Supramarginal gyrus (BA40) | -0.05 | 0.00 | 0.20 | 0.23 | 0.23 | 0.44 | 0.36 | 0.46 | 0.34 | 0.41 | 0.48 | 0.79 | 0.63 | 0.55 | 0.31 | 0.69 | 0.78 | 0.87 | 0.65 | 0.74 | -0.04 | 0.07 | **0.11** |
| Pars opecularis (BA44) | -0.05 | -0.03 | 0.05 | 0.19 | 0.20 | 0.34 | 0.25 | 0.55 | 0.34 | 0.34 | 0.48 | 0.81 | 0.77 | 0.65 | 0.53 | 0.68 | 0.76 | 0.63 | 0.66 | 0.85 | -0.06 | 0.05 | **0.04** |
| Superior parietal lobule (BA7) | 0.09 | 0.09 | 0.23 | 0.35 | 0.29 | 0.56 | 0.57 | 0.57 | 0.50 | 0.57 | 0.70 | 1.00 | 0.92 | 0.78 | 0.53 | 1.04 | 1.18 | 1.00 | 0.81 | 0.97 | -0.02 | 0.09 | **0.15** |
| Ventro-medial prefrontal cortex (BA 11–15, 25) | -0.10 | -0.03 | 0.10 | 0.16 | 0.18 | 0.25 | 0.33 | 0.53 | 0.41 | 0.52 | 0.40 | 0.94 | 0.54 | 0.86 | 0.31 | 0.57 | 0.71 | 0.64 | 0.62 | 0.87 | -0.10 | 0.07 | **0.03** |
| Ventral anterior cingulate cortex (BA24) | 0.12 | 0.16 | 0.25 | 0.32 | 0.32 | 0.47 | 0.47 | 0.57 | 0.48 | 0.56 | 0.51 | 1.04 | 0.92 | 0.71 | 0.59 | 0.86 | 0.68 | 0.64 | 0.64 | 0.91 | 0.06 | 0.08 | **0.23** |
| Pars triangularis (BA45) | -0.10 | -0.02 | 0.07 | 0.19 | 0.27 | 0.32 | 0.34 | 0.65 | 0.43 | 0.43 | 0.49 | 1.00 | 0.68 | 0.85 | 0.43 | 0.72 | 0.68 | 0.75 | 0.64 | 0.88 | -0.08 | 0.05 | **0.02** |
| Inferior prefrontal gyrus and pars orbitalis (BA47) | -0.07 | -0.07 | 0.10 | 0.12 | 0.25 | 0.16 | 0.33 | 0.49 | 0.37 | 0.48 | 0.38 | 0.85 | 0.70 | 0.66 | 0.25 | 0.42 | 0.56 | 0.54 | 0.58 | 0.68 | -0.12 | 0.06 | **0.00** |
| Superior dorsolateral prefrontal cortex (BA9) | -0.03 | 0.19 | 0.28 | 0.26 | 0.18 | 0.57 | 0.40 | 0.76 | 0.56 | 0.41 | 0.58 | 1.13 | 0.78 | 1.04 | 0.60 | 0.79 | 0.81 | 0.90 | 0.58 | 1.27 | -0.02 | 0.08 | **0.15** |
| Anterior prefrontal cortex (BA10) | -0.08 | 0.16 | 0.27 | 0.34 | 0.21 | 0.43 | 0.54 | 0.76 | 0.58 | 0.63 | 0.57 | 1.33 | 0.78 | 1.05 | 0.49 | 0.95 | 0.83 | 1.01 | 0.66 | 1.28 | -0.04 | 0.08 | **0.11** |
| Dorsal anterior cingulate cortex (BA32) | 0.05 | 0.21 | 0.35 | 0.35 | 0.30 | 0.67 | 0.53 | 0.73 | 0.64 | 0.68 | 0.66 | 1.34 | 0.90 | 0.94 | 0.77 | 0.97 | 0.90 | 0.90 | 0.78 | 1.25 | 0.03 | 0.08 | **0.19** |
| Inferior dorsolateral prefrontal cortex (BA46) | -0.08 | 0.20 | 0.24 | 0.34 | 0.25 | 0.42 | 0.44 | 0.67 | 0.59 | 0.50 | 0.57 | 1.14 | 0.82 | 0.95 | 0.40 | 0.76 | 0.81 | 1.00 | 0.58 | 1.11 | -0.05 | 0.06 | **0.07** |
| Striatum | 0.29 | 0.40 | 0.41 | 0.36 | 0.56 | 0.49 | 0.89 | 0.59 | 0.70 | 0.40 | 0.75 | 0.61 | 0.64 | 0.81 | 0.67 | 0.94 | 0.99 | 0.66 | 0.76 | 0.74 | 0.02 | 0.07 | **0.16** |

**Supplementary Figure 1~~2~~ The regional non–displaceable binding potential (BP_ND_) data used for the determination of amyloid positive extra–striatal regions in the PIB–positive group, together with regional group–wise data for the PIB–negative group.**

The columns in the shaded area represent individual participants (P1–P20) in the PIB–positive group (n=20), as defined by their striatal BP_ND_ (see Figure 1). The rows represent each region of interest. Mean and standard deviation (Stdev) of the regional BP_ND_ data for PIB–negative participants (n=29) were calculated and are shown in the unshaded columns on the right. Shaded cells represent regions for PIB–positive participants that have a BP_ND_ value above the mean + 2 Stdev of the PIB–negative group and hence are classified as amyloid positive. Amyloid positive regions in the PIB–positive group were used for the creation of the PIB staging model that is presented in Figure 3. Dark coloured cells represent amyloid positive regions consistent with the staging model, while lightly coloured cells denote amyloid positive regions that did not satisfy all the conditions of this model.
